# Case Report: Primary leiomyosarcoma of the breast

**DOI:** 10.3389/fonc.2025.1571029

**Published:** 2025-05-22

**Authors:** Haoqiang Lv, Haiyang Ren, Shuang Zhang, Gen Ding, Ping Tian

**Affiliations:** ^1^ Department of General Surgery, Beidahuang Group Hongxinglong Hospital, Shuangyashan, Heilongjiang, China; ^2^ Department of General Surgery, Linyi Maternity and Child Health Care Hospital, Linyi, Shandong, China; ^3^ Department of Pathology, Beidahuang Group Hongxinglong Hospital, Shuangyashan, Heilongjiang, China; ^4^ Department of Medical Imaging, Linyi Maternity and Child Health Care Hospital, Linyi, Shandong, China; ^5^ Department of Gastroenterology, Linyi People’s Hospital, Linyi, Shandong, China

**Keywords:** breast, primary leiomyosarcoma, sarcoma, breast malignancy, mastectomy

## Abstract

Primary leiomyosarcoma of the breast is an extremely rare tumor originating from the mesenchymal tissue of the breast. The clinical data of a case of primary leiomyosarcoma of the breast admitted to our hospital on September 11, 2024 were analyzed. A 58-year-old woman had a right breast mass for a week. Physical examination revealed an irregular mass at approximately 2–3 points in the right upper quadrant of the breast. Laboratory tests showed normal results for the tumor marker CA153. Color Doppler ultrasound considered solid nodules (BI-RADS Class 4b). Modified radical mastectomy and tertiary axillary lymph node dissection was performed on 2024-9-13, and the patient underwent adjuvant chemotherapy after surgery and recovered well. Pathological findings were reported one week after surgery: breast leiomyosarcoma. This article presents the diagnosis and treatment of primary leiomyosarcoma of breast through a clinical case, which provides a reference for clinicians. This case highlights the importance of pathological examination in the diagnosis of rare tumors, and suggests the need to combine imaging, laboratory examination and pathological findings to make a comprehensive judgment of breast masses.

## 1 Introduction

Primary breast sarcoma is a rare tumor, accounting for less than 1% of all breast malignancies and less than 5% of all soft tissue sarcomas (1–3), while primary leiomyosarcoma of breast is an extremely rare subtype of breast sarcoma, accounting for less than 0.0006% of all breast malignancies. Fewer than 80 cases have been reported in the literature so far (4–6). There are even fewer reports in medical journals. At present, no specific treatment plan has been determined. To determine the appropriate diagnosis and treatment management plan, we evaluated this case and reviewed relevant literature to discuss its diagnosis, treatment and prognosis.

## 2 Case description

### 2.1 Patient information

A 58-year-old woman presented with “a right breast mass for a week.” A week ago, the patient inadvertently found an irregular tumor on the right breast, about the size of an egg, without redness, swelling or pain, nipple discharge or nipple invagination, and normal skin on the surface of the tumor. Previously in good health. Menopause about 5 years.

### 2.2 Clinical findings

Physical examination: An irregular mass, about 4.0 cm × 2.0 cm × 2.0 cm in size, was visible at about 2–3 points in the upper quadrant of the right inner breast about 1.0cm from the areola, with tough quality, clear boundary, moderate motion, and no tenderness. A 1.0 cm × 1.0 cm circular mass can be found in the right axilla. Lymph node enlargement is considered.

### 2.3 Timeline

The patient had no family or significant past history of breast cancer.

### 2.4 Diagnostic assessment

**Figure 1:**
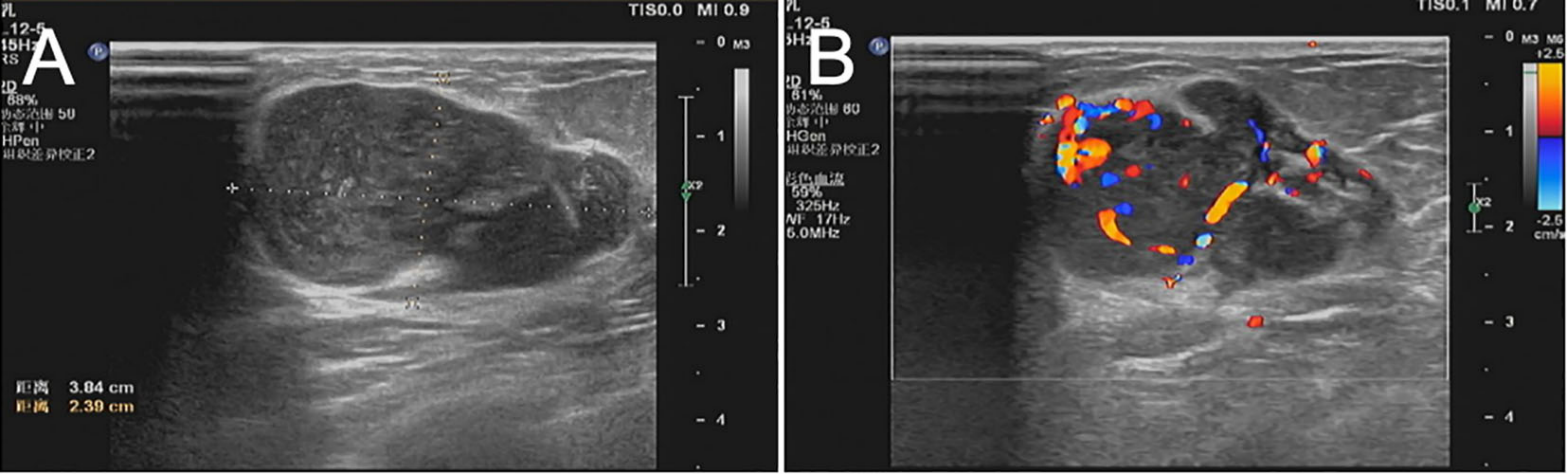
**(A, B)** Ultrasonography showed that the hypoechoic nodules of 3.8 cm × 2.3 cm × 2.8 cm in size were visible at about 2–3 points in the upper quadrant of the right mammary gland, with clear boundaries, less uniform internal echo, irregular shape, lobed shape, and rich blood flow signals could be detected. It was classified via ultrasonography in Breast Imaging-Reporting and Data System (BI-RADS 4b) category.

**Figure 2:**
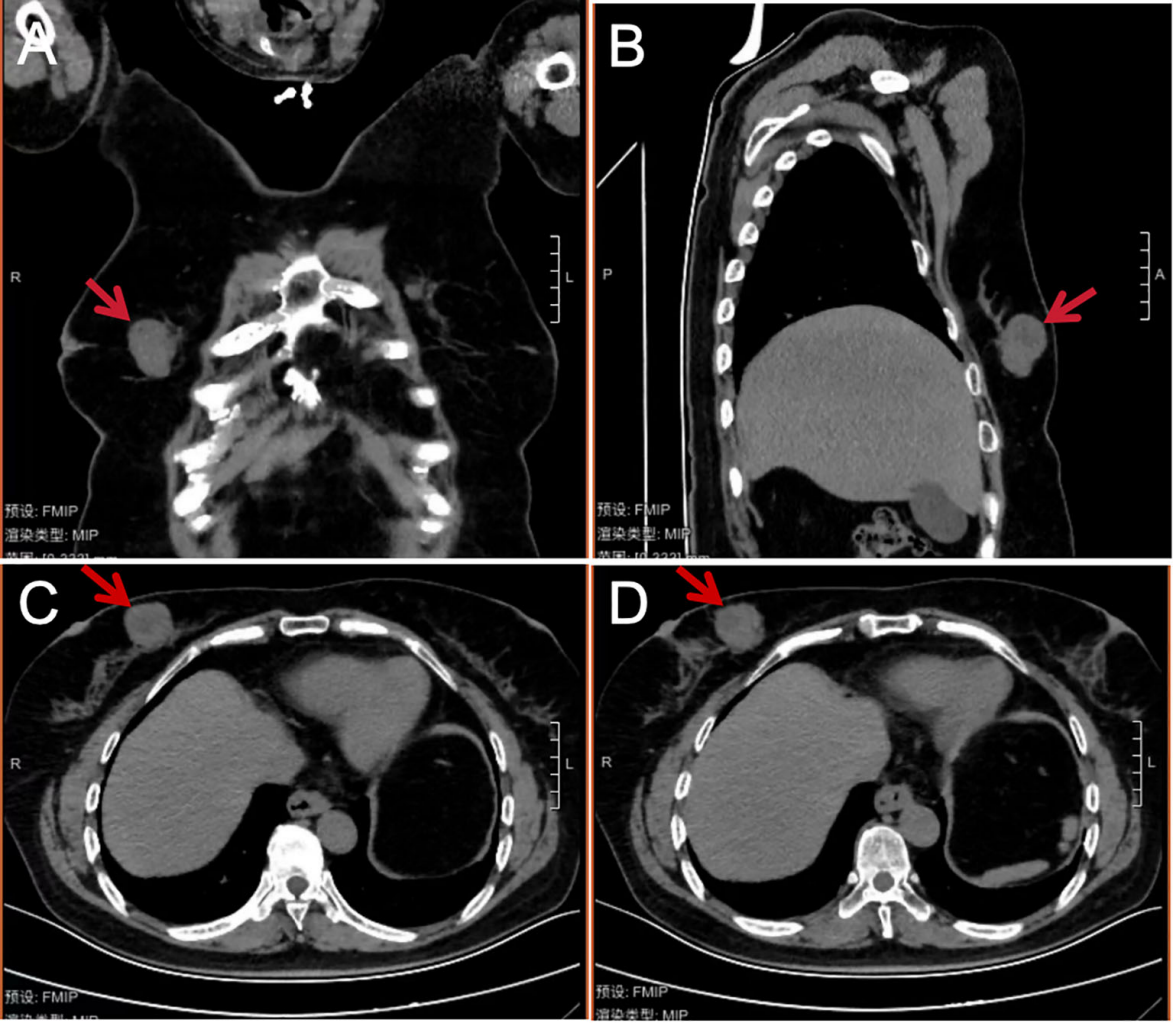
Coronal **(A)**, sagittal **(B)**, and cross-sectional **(C, D)** chest CT images of the patient in mediastinal window view before breast tumor resection. A soft tissue mass (shown by the red arrow at about 3 points) can be seen on the medial side of the right mammary gland. The density is not uniform, the shape is not regular, the lobulated mass can be seen, the size is about 4.0 cm × 2.0 cm × 2.0 cm, the CT value is about 17 HU, and the boundary is clear.

B-ultrasound examination showed that a low-echo nodule of about 3.8 cm × 2.3 cm × 2.8 cm in size was visible at about 2–3 points in the upper quadrant of the right mammary gland, with clear boundaries, uneven internal echo, irregular shape, lobed shape, and rich blood flow signals could be detected. Multiple lymph node echoes can be seen in the right axilla, with clear boundary of skin and pulp, the larger one is about 1.6 cm × 0.68 cm. Ultrasound results showed solid nodules in the upper quadrant of the right internal breast (BI-RADS 4b) (**Figure 1**). Preoperative completed lung CT showed that there was a tumor on the right breast, about 4.0 cm × 2.0 cm× 2.0 cm in size, with a CT value of about 17 HU, and the boundary was acceptable (**Figure 2**). Laboratory examination results: Blood routine: WBC 8.26 × 10^9/^L, tumor marker CA153 5.98 U/mL. Typically, these patients do not have abnormal serological values or tumor markers, as was the case in this case (7).

### 2.5 Therapeutic intervention

After improving the preoperative examination, the patient met the surgical indications and had no obvious contraindications. The patient underwent surgical treatment on the third day after admission and underwent resection of breast masses. The rapid pathological return during the operation was malignant tumor of the mesenchymal tissue of the breast. Combined with the preoperative ultrasound report of the patient, multiple swollen lymph nodes in the right axilla were evaluated, and modified radical mastectomy and tertiary axillary lymph node dissection were performed. Ensure that the width of the cutting edge is at least 3 cm. Two indwelling drainage tubes.

### 2.6 Follow-up and outcomes

**Figure 3:**
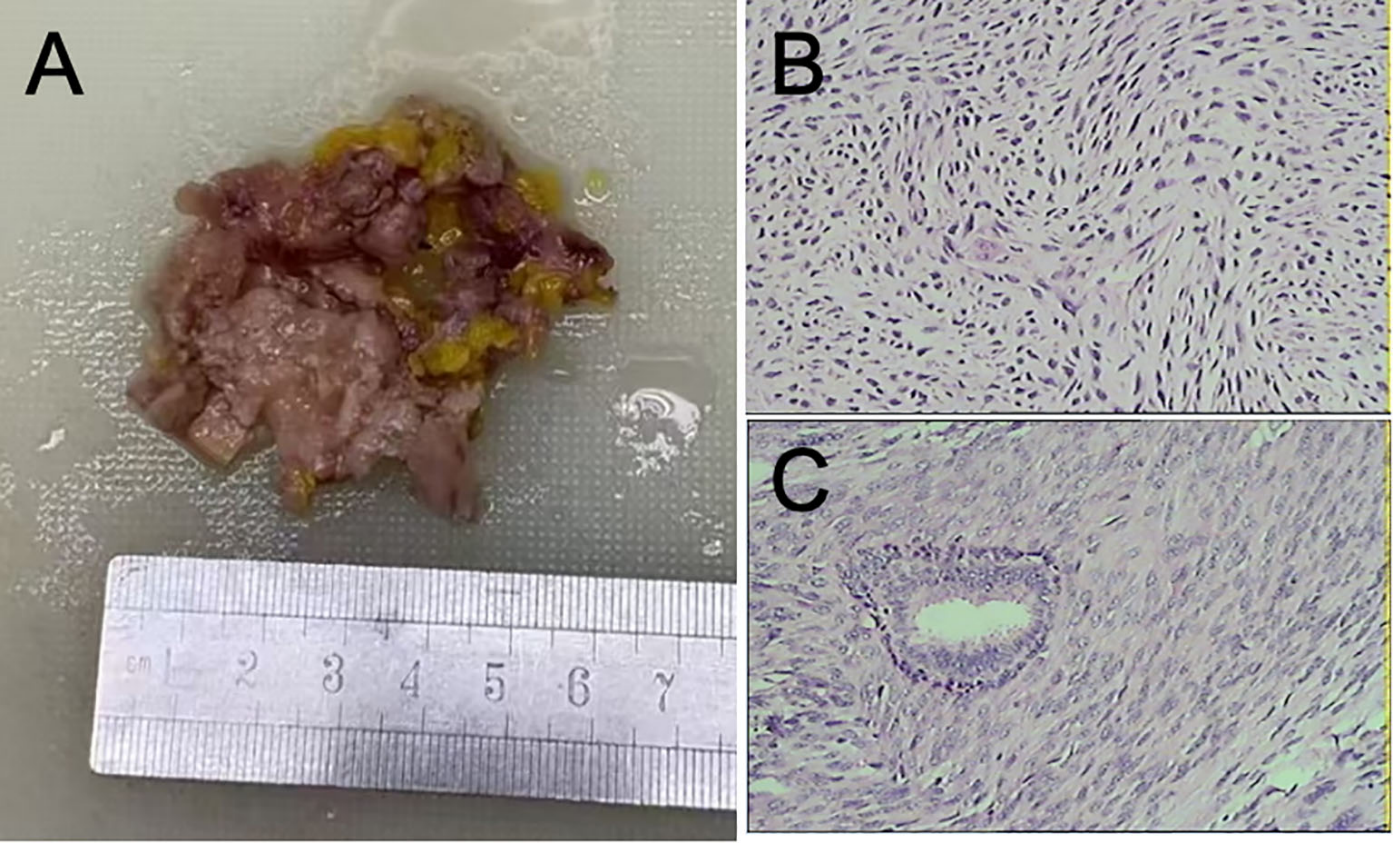
**(A)** Right breast mass, volume: 6.0 × 5.0 × 1.2 cm^3^, section gray, intraoperative freeze pathology. **(B, C)** Photomicrographs of the tumor. The tumor cells were arranged in parallel, fascicular interlace, accompanied by mucoid degeneration and more nuclear division. Pathological diagnosis: right breast leiomyosarcoma, highly differentiated, tumor volume 5.0 cm × 2.0 cm × 1.0 cm^3^, accompanied by mucinous degeneration, mitotic image 24/10 HPE, no clear invasion of vascular and divine realm. Right subaxillary lymph node 0/26 (HE, 40×).

Conventional treatment was given after surgery. Postoperative pathological findings: The tumor cells were arranged in parallel, fascicular-like interwoven arrangement, accompanied by mucoid degeneration and more nuclear division. The tumor section was grayish white, and the nipple and incisal margin were negative. Diagnosis: Right breast leiomyosarcoma, highly differentiated tumor volume 5.0 cm × 2.0 cm × 1.0 cm, accompanied by mucinous degeneration, nuclear fission image 24/10 HPE, no clear invasion of vascular and divine realm. Right subaxillary lymph node 0/26 (–) (**Figure 3**).

**Figure 4:**
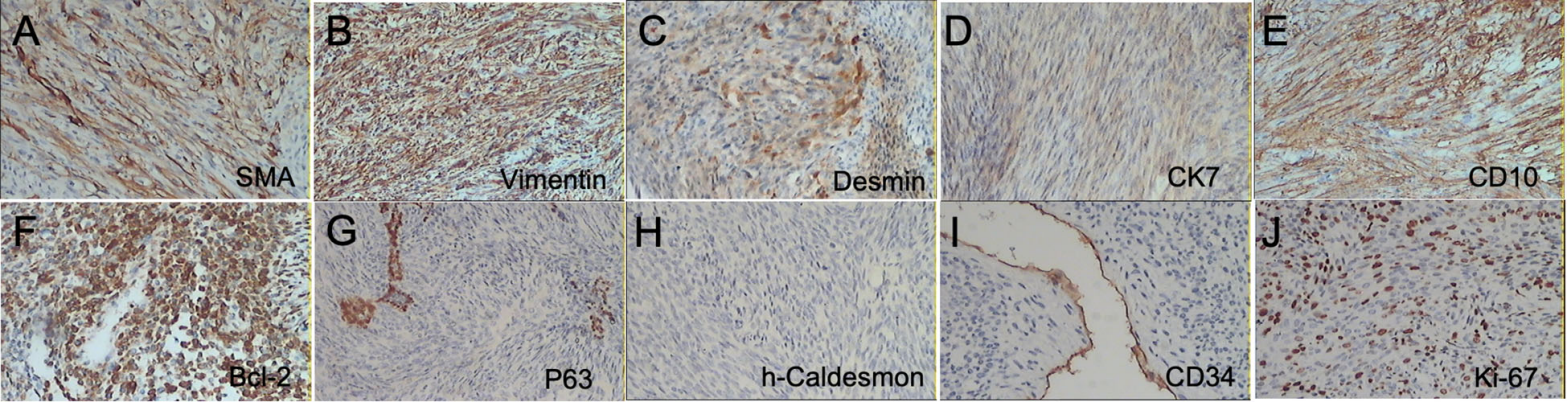
Immunohistochemical staining was positive for SMA, Vimentin, Desmin, CK7, CD10, and Bcl-2 **(A–F)** and negative for P63, h-Caldesmon, and CD34 **(G–I)**, respectively. **(J)** Ki67 -30% (10×).

Given the morphological diagnosis of sarcoma, a large immunohistochemistry (IHC) panel was applied to reach a definitive diagnosis (**Table 1**). Vimentin, Desmin, and CD10 were strongly positive in tumor cells, and SMA, CK7, and Bcl-2 showed patchy strong positivity. P63, h-Caldesmon, and CD34 were negative. The Ki67 proliferation index was 30% (**Figure 4**). The clinical stage of this case follows the standard TNM stage of breast malignancy (6, 8), which is T2N0M0.

**Table 1:** Immunohistochemistry panel.

Marker	Clone	Interpretation	Marker	Clone	Interpretation
Vimentin	V9	Positive	Bcl-2	SP66	Positive
Desmin	D33	Positive	P63	4A4	Negative
CD10	56C6	Positive	h-Caldesmon	h-CALD	Negative
SMA	1A4	Positive	CD34	QBEnd-10	Negative
CK7	C35	Positive	Ki-67	MIB7	30%

Postoperative follow-up was conducted on the patient, and the patient recovered well after surgery. The drainage tube was removed and stitches were removed on the 15th day after surgery. There was no dysfunction or numbness in the affected upper limb, and the muscle strength was normal. Chemotherapy is recommended.

## 3 Discussion

Breast leiomyosarcoma is an uncommon tumor. They may originate from smooth muscle cells or stromal mesenchymal cells in the lining of blood vessels. In histopathological evaluation, immunohistochemical staining as an adjunct is essential to distinguish leiomyosarcoma from other tumors and soft tissue sarcomas. These tumors are usually Desmin and SMA positive and S100 and CKpan negative (6, 9–11). In the treatment options for primary leiomyosarcoma, surgery is generally considered to be the main treatment option (12). Previous studies recommend at least 3 cm negative incisal margin for optimal results (4, 9, 13). Since there have been no reported cases of lymphatic metastasis via hematogenous route, axillary lymph node dissection is not necessary if leiomyosarcoma can be confirmed before or during surgery (6, 8, 9, 14, 15). In this case, lymph node enlargement was considered by preoperative ultrasound, and metastasis could not be clearly ruled out, so lymph node dissection was performed. So we did not use a wide local excision. However, postoperative pathology reported that lymph nodes showed increased reactivity and no metastasis. Such diseases are often further treated with radiotherapy and chemotherapy after surgery. The patient went to the oncology department for chemotherapy after surgery. AC chemotherapy regimen, including doxorubicin and cyclophosphamide, was administered every 21 days for a total of 4 courses. Nevertheless, the patient did not complain of discomfort, and there were no adverse and unanticipated events during the treatment. The patient had been followed up for six months, and no recurrence or metastasis had been detected. Long-term follow-up of this disease is important because a proportion of these tumors have been reported to recur after treatment (2, 16). We reviewed the cases reported in the English literature in the past 10 years, as shown in **Table 2**.

**Table 2:** Literature review describing characteristics and treatment of primary leiomyosarcomas.

Case	Author/year	Location	Age/Sex	Size (cm)	Ultrasound features	CT	Treatment	Local recurrence	Metastasis	Outcome and follow-up
1	Yang FY et al/2024 (17)	Left/outer quadrant of the left breast	78/F	2.5	Hypoechoic; BI-RADS 4a	Middle density of space-occupying lesion; The CT value was about 27 HU	Resection of malignant breast tumor with left breast reserved	No	No	Alive, 36 months
2	Sethi E et al/ 2024 (2)	Left/upper and lower outer quadrants	37/F	17	–	–	Mastectomy	Yes	Lung	Death after 3 months
3	Samenova D et al /2023 (3)	Right/upper outer quadrants	45/F	21	–	Space-occupying lesion	Total right mastectomy+ axillary lymph node dissection	No	No	Alive, 60 months
4	Masadah R et al/ 2023 (9)	Left	30/F	12	Hypoechoic;BI-RADS 5	–	Wide local excision	No	No	Alive, 8 months
5	Ely Cheikh T et al /2021 (13)	Left/lower inner quadrant	65/M	5.3	Hypoechoic	–	Wide local excision	No	No	Alive, 11 months
6	Galama R et al /2020 (12)	Left/lower quadrant	87/F	8	Polycyclic contours and surrounding oedema, with cutaneous thickening; BI-RADS 4	–	Excision + radiotherapy	No	No	Alive, 20 months
7	Horton L et al /2020 (4)	Right/upper outer quadrants	61/F	1.6	Hypoechoic	–	wide local excision and planned cosmetic breast reduction surgery	–	–	Alive, 6 months
8	Xu Z et al /2019 (18)	Left	51/F	–	–	–	Excision	Yes	No	Alive, 9 months
9	Miyazaki C et al /2019 (5)	Left	52/F	10	–	Enlarged axillary lymph nodes	Total left mastectomy+ axillary lymph node dissection+neoadjuvant chemotherapy	No	No	Alive, 18 months
10	Félix C et al /2019 (11)	–	84/F	5.5	Hypoechoic	–	Excision	Yes	Liver, gallbladder, and pancreatic	Alive, 72 months
11	Lee SY et al /2018 (16)	Left	49/F	8	–	Large, lobulated, heterogeneously enhancing mass	Palliative total mastectomy of the left breast+palliative chemotherapy	Yes	Lung	Death after 6 months
12	Amberger M et al/2018 (6)	Left/lower quadrants	20/F	3	Likely hematoma	–	Total left mastectomy+axillary lymph node dissection	Yes	Lung	Alive, 32 months
13	Brandão RG et al/2017 (19)	Left/upper quadrants	68/F	1.8	Hypoechoic; BI-RADS 4.	–	Excision	No	No	Alive, 60 months
14	Testori A et al /2017 (7)	Left/retroareolar region of the breast	62/F	3.5	Oval shape, well-defined margins and slightly heterogeneous echostructure	–	Excision + radiotherapy	–	–	–
15	Tajima S et al /2015 (20)	Left/central localization	50/F	4.8	Cystic appearance	–	Total left mastectomy	No	No	Alive, 6 months
16	Kim et al /2015 (21)	Right/upper outer quadrant	51/F	0.5	Oval, hypoechoic mass with no vascularity in the subcutaneous fat layer	–	Excision + chemotherapy	No	No	Alive, 60 months

F, female; M, male; “-” indicating information not found within the report.

## 4 Conclusion

Primary Leiomyosarcoma of the Breast is an extremely rare malignant tumor of the breast, usually originating in blood vessels, papillary smooth muscle or leiomyoma malignant transformation, mainly through blood metastasis, local lymph node involvement is rare. Pathological examination is the key to diagnosis, typical manifestations are spindle cells, nuclear atypia, and more mitotic signs, and the expression of immunohistochemical markers such as SMA and Desmin is helpful to distinguish from other tumor types.

Surgical resection is the primary treatment, with a negative margin and usually without axillary lymph node dissection. The role of chemotherapy and radiotherapy is unclear and needs to be evaluated on an individual basis. Although the prognosis is superior to other breast sarcomas, the risk of local recurrence and distant metastasis still requires long-term follow-up. At present, there is no uniform diagnosis and treatment standard, and the prognostic factors are not fully defined. Since the cases are rare and mostly reported as individual cases, further accumulation and analysis of patient data are needed to optimize treatment strategies, clarify disease characteristics, and provide a more powerful guidance basis for clinical practice.

## Data Availability

The original contributions presented in the study are included in the article/supplementary material. Further inquiries can be directed to the corresponding author.
